# Effects of dietary energy levels on microorganisms and short-chain fatty acids of rumen and tight junction proteins in Honghe Yellow cattle

**DOI:** 10.3389/fmicb.2024.1335818

**Published:** 2024-04-02

**Authors:** Ye Yu, Yujie Zi, Runqi Fu, Binlong Fu, Chenghuan Li, Yaqi Lv, Zhe Li, Huayu Wang, Jing Leng

**Affiliations:** ^1^Faculty of Animal Science and Technology, Yunnan Agricultural University, Kunming, China; ^2^Key Laboratory of Animal Nutrition and Feed Science of Yunnan Province, Yunnan Agricultural University, Kunming, China

**Keywords:** Honghe Yellow cattle, rumen microbiota, short chain fatty acids, tight junction proteins, energy levels

## Abstract

This study was conducted to investigate the effects of dietary energy levels on microorganisms and short-chain fatty acids (SCFAs) of rumen and the expression of tight junction proteins in Honghe Yellow cattle. A total of fifteen male Honghe Yellow cattle were randomly divided into three treatments (five replicates per treatment), consisting of formulated energy concentrations of 5.90 MJ/kg (high-energy diet, group H), 5.60 MJ/kg (medium-energy diet, group M) and 5.30 MJ/kg (low-energy diet, group L). The results showed that compared with group H, the expression of Claudin-1 in rumen epithelium of groups M and L was increased, but the expression of ZO-1 was decreased (*p* < 0.05). Moreover, compared with group H, group M down-regulated the expression of Occludin and Claudin-1 in the brain (*p* < 0.05). For rumen bacteria, the dominant phyla included Bacteroidetes and Firmicutes, the abundance of Actinobacteriota in groups M and L was significantly increased compared with group H (*p* < 0.05). At the genus level, the relative abundance of *Corynebacterium*, *Eubacterium_nodatum_group* and *Neisseraceae* in groups M and L was significantly decreased compared with group H (*p* < 0.05). For rumen fungi, the dominant phyla included Basidiomycota, Ascomycota and Neocariastigomycota, the relative abundance of Ascomycetes was significantly higher than that of groups M and L compared with group H (*p* < 0.05). At the genus level, the relative abundance of *Neocelimastigaceae* and *Myceliophthora* in groups M and L was significantly reduced compared with group H (*p* < 0.05). Furthermore, the expression of Claudin-1 in rumen epithelium was significantly positively correlated with *Actinobacteriota*, *Corynebacterium* and *Neisseriaceae.* The expression of ZO-1 in the spinal cord was significantly positively correlated with *Myceliophthora*. The expression of Occludin in brain was positively correlated with valerate content (*p* < 0.05). In summary, dietary energy levels affected the rumen microbiota of Honghe Yellow cattle. The expression of Claudin-1 in rumen epithelium and the total SCFAs concentration were increased with decreasing dietary energy levels, but the expression of Claudin-1 in brain and ZO-1 in the spinal cord were reduced with decreasing dietary energy levels. Meanwhile, the rumen microbiota and SCFAs were significantly correlated with the expression of TJP.

## 1 Introduction

Rumen microbiota participates in the metabolism of the host and exchanges materials and information. Rumen microbiota had a strong symbiotic relationship with microorganisms and co-evolved with the host (Faust and Raes, 2012; Lin et al., 2019). Rumen microbiota can help hosts break down substances such as cellulose and hemicellulose that cannot be directly utilized by the host, thus providing energy and nutrition to the host (Hooper et al., 2002). Bacteria are the most abundant microorganisms in the rumen, which can digest fiber, starch, protein and other nutrients required by the host (Huws et al., 2018). Meanwhile, rumen fungi, as a “biological crowbar,” can not only mechanically Pierce the plant cell wall through the root, but also cooperate with rumen bacteria. Various enzymes such as highly active cellulase and hemicellulase are secreted to chemically act on some plant tissues that are difficult to degrade, both of which play an important role in the process of forage fermentation and host energy supply (Kumar et al., 2013).

Alterations in the structure of rumen microbiota can affect rumen digestion and metabolism as well as production performance. The composition of rumen microbiota is influenced by various factors such as diet, species, physiology and environment, among which diet is the most important influencing factor (Lin et al., 2018). Noteworthy, the rumen microbiota plays an important role in feed digestion for cattle. The dietary energy level can affect the rumen microbiota, which can influence gastrointestinal peristalsis and participate in the digestion and utilization of the diet. For the most part, the diversity and abundance of rumen microbes increased with decreasing food energy levels (Wang et al., 2021). It was reported that the high-energy diet increased the number of ruminal amylolytic bacteria and propionate-producing bacteria, such as *Prevotella, Amylobacter amylophilus*, *Amylosuccinomonas*, and *Bifidobacterium* (Hu et al., 2020). Similarly, it was found the low energy level diet increased the relative abundance of Fibrinobacteria, and at the genus level, the relative abundance of *Vibrio butyricum* and *Prevotella*, significantly decreased the relative abundance of Rikenellaceae (Matsushita et al., 2021). These results indicated that the rumen bacterial dominant genera were dramatically influenced by the energy level of the diet (Park et al., 2020). The studies have shown that the relative abundance of *saccharomyces cerevisiae* in high energy diets is higher than that on low energy diets (Park et al., 2020). Furthermore, the rumen of ruminants is a natural fermentation tank, in which there are all kinds of microorganisms. Short chain fatty acids (SCFAs) produced by specific gastrointestinal microbiota fermentor feed are the bridge of interaction between microbiota and host (Van der Hee and Wells, 2021). More than 95% of the SCFAs in the rumen is acetate, propionate, and butyrate, while the content of isobutyrate, valerate, and isovalerate is rare (Parada Venegas et al., 2019). Previous study found that dietary energy level can significantly increase the proportion of propionate, reduce the proportion of acetate and isobutyrate, and the A/P ratio (Wang et al., 2020). Research showed that the feed with high energy levels reduced the content of acetate and increased the content of propionate (Xie et al., 2021). Overall, there have been relatively more studies on dietary energy affecting rumen bacteria, but fewer studies on rumen fungi, moreover, there is no relevant study on the effect of dietary energy level on microorganisms and SCFAs in Honghe Yellow cattle. And the effect of dietary energy on the contents of SCFAs in the rumen of Honghe Yellow cattle is incomplete.

Communication from the gut microbiome to the central nervous system (CNS) occurs primarily through microbial derivatives, which include SCFAs, secondary bile acids and tryptophan metabolites (Wikoff et al., 2009). SCFAs have a mediating role in microbiota-gut-brain axis crosstalk (Dalile et al., 2019). Fermentation of dietary carbohydrates by the gut microbiota produces SCFAs, which can cross the blood–brain barrier (BBB), flow through brain tissue, and are involved in the regulation of gut motility function and blood–brain barrier permeability, thereby affecting the expression of tight junction proteins (TJP) (Sleeth et al., 2010; Frost et al., 2014; Wishart et al., 2018). TJP are crucial form of intercellular junction and the most significant structure of mucosal mechanical barrier that is composed of Claudin protein, Occludin protein, ZOs and other structural proteins and various connexin molecules. TJP can mediate paracellular permeability and is responsible for intercellular transport and intestinal barrier permeability (Kim et al., 2018). Claudins are the most abundant transmembrane transport protein in TJP, which plays an important role in maintaining the permeability (Tsukita et al., 2019). Claudins are expressed in all epithelial tissues, and various Claudins are expressed at the same time, for example, Claudin-1 can be identified in the liver, brain, intestine, skeletal muscle, kidney, and other organs and tissues (Zihni et al., 2016). Occludin is the first complete membrane TJP identified, and no homolog was found (Cummins, 2012). ZO-1 is the cytoplasmic component of TJP and adhesive junction protein (Fanning et al., 2002). Previous studies indicated that energy is one of the important factors affecting TJP expression in animal tissues (Cui et al., 2020).

Consequently, considering the above, we postulated that dietary energy levels have the potential to affect the microorganisms and SCFAs of rumen and tight junction proteins of rumen and brain. In this study, representative cattle breeds in China (Honghe Yellow cattle) were used as experimental animals, which demonstrated these hypotheses by assessing the effects of different dietary energy levels (high, medium, and low energy) on rumen microbiota, SCFAs in rumen fluid and TJP in tissues of Honghe Yellow cattle.

## 2 Materials and methods

### 2.1 Animals and sampling

A total of fifteen male Honghe Yellow cattle with similar body weight (231.9 ± 7.87 kg) and age (2 years old) were randomly divided into three groups and fed with high-energy (5.90 MJ/kg), medium-energy (5.60 MJ/kg) and low-energy (5.30 MJ/kg) diets, respectively (Table 1). Cattle were fed individually twice a day and allowed free access to water. The pre-experiment period was 14 days and the experiment period was 70 days. At the end of feeding experiment, three cattle were randomly selected from each group to be slaughtered. The rumen fluid was then collected for analysis of rumen microbiota and short-chain fatty acids (SCFAs) concentration. In addition, brain, cerebellum, spinal cord and rumen epithelial tissues were collected for determination of junction proteins (TJP) (Occludin, Claudin-1 and ZO-1) expression. The collected rumen fluid and tissue samples were stored at −80°C.

**Table 1:** Diet composition and nutrient levels (dry matter).

Items	Group H	Group M	Group L
Composition			
Corn silage %	49.0	53.0	58.0
concentrate supplement %	41.3	40.5	36.0
CP powder %	4.2	1.5	2.0
Fat powder %	5.5	5.0	4.0
Total %	100	100	100
Nutrient levels			
NEmf MJ/kg	5.90	5.60	5.30
CP %	11.72	11.74	11.73
Ca %	0.59	0.58	0.55
P %	0.63	0.62	0.64
NDF %	40.63	43.64	46.27
ADF %	26.55	28.77	30.80

The concentrate supplement: soybean meal 12%, corn gluten meal 9%, distiller's grains 5%, corn 63%, bran 6%, Dicalcium phosphate 3%, NaCl 1%, premix 1%; Concentrated feed supplement is provided per kilogram of feed: vitamin A 64 000 IU, vitamin D 19 150 IU, vitamin E 80 mg, Cu 36 mg, Fe 45 mg, Mn 24 mg, Zn 33 mg, Co 0.10 mg, I 33 mg, Se 0.28 mg. The comprehensive net energy is the calculated value, and the rest of the nutritional indicators are the actual-measured values.

### 2.2 Determination of the expression of TJP

#### 2.2.1 RNA extraction and reverse transcription

Total RNA was extracted from rumen epithelium, brain, cerebellum, and spinal cord by the Trizol method (Invitrogen, Carlsbad, CA, U.S.). The RNA integrity was checked by gel electrophoresis. The extracted RNA was reverse transcribed into cDNA according to the instructions of the reverse transcription kit and stored at −20°C. Primer sequences for the target genes were shown in Table 2.

**Table 2:** Primer sequence.

Genes	Primer (5´→3´)	Source	Size (bp)	Tm (°C)
Claudin-1F	CGTGCCTTGATGGTGAT	NM-001001854	102	52.3
Claudin-1R	CTGTGCCTCGTCGTCTT			
Occludin-F	GAACGAGAAGCGACTGTATC	NM-001082433	122	53.8
Occludin-R	CACTGCTGCTGTAATGAGG			
ZO-1F	TCTGCAGCAATAAAGCAGCATTTC	XM-010817146	187	55.2
ZO-1R	TTAGGGCACAGCATCGTATCACA			
GAPDH-F	GGGTCATCATCTCTGCACCT	NM-001034034.2	176	55.8
GAPDH-R	GGTCATAAGTCCCTCCACGA			

#### 2.2.2 Fluorescent quantitative PCR

SYBR was used as a fluorescent dye and TB Green produced by TaKaRa^®^ Premix Ex Taq™ II (Tli RNaseH Plus) conducts qRT-PCR reaction according to the reaction system. PCR amplification conditions: pre-denaturation at 95°C for 30 s, denatured at 95°C for 5 s, annealed at 60°C for 30 s, extended at 72°C for 20 s, cycled for 39 times, and fluorescence collection was selected after extension. After the reaction, the dissolution curve was analyzed to determine the specificity of the qRT-PCR reaction. The calculation method of 2^−△△Ct^ was used for relative quantitative analysis.

#### 2.2.3 Data statistics and analysis

All data were analyzed by one-way ANOVA using SPSS 25 statistical software. Values were expressed as means ± SEM. *p* < 0.05 was considered statistically significant unless otherwise stated. Finally, the data of rumen microbiota, SCFAs, and TJP were analyzed by using the Pearson correlation analysis.^1^

### 2.3 16S Rdna and its sequencing

#### 2.3.1 DNA extraction

2 mL of rumen samples were used to extract microbial DNA using the kit method (Omega Bio-tek, 120 Norcross, GA, U.S.).

#### 2.3.2 PCR amplification

The purity and concentration of DNA were detected by 1.0% agarose gel electrophoresis and NanoDrop2000, and an appropriate amount of the sample was diluted in a centrifuge tube, followed by PCR amplification. Diluted DNA was used as a template. The bacteria were sequenced with 16S rRNA, and the amplified region was a 338F-806R primer of V3 + V4 region, with a length of 468 bp. The upstream sequence was ACTCCTACGGGGGGCAGCAG, and the downstream sequence was GGACTACHVGGGTWTCTAAT. The fungi were sequenced by Internal Transcribed Spacer (ITS), the amplified region was ITS1F-ITS2R, with a length of 350 bp. The upstream was CTTGGTCATTTAGAGGAAGTAA, and the downstream was GCTGCGTTCTTCATCGATGC.

The amplification system (20 μL): 5 × Trans Start Fast Pfu buffer 4 μL, DNTPs (2.5 mM)2 μL, Trans Start Fast Pfu Polymerase 0.4 μL, BSA 0.2 μL, 338F (5 μM)0.8 μL, 806R(5 μM)0.8 μL, Template DNA 10 ng, make up ddH2O to 20 μL. 3 replicates per sample. PCR amplification procedure: pre-denaturation at 95°C for 3 min, denaturing at 95°C for 30 s, annealing at 55°C for 30 s, and stretching at 72°C for 30 s, with 30 cycles, then it was extended at 72°C for 10 min, and finally, it was stored at 4°C.

#### 2.3.3 Mixing and purification of PCR products

Mixed the samples with equal concentration according to the detection results of the concentration of PCR products. After full mixing, used agarose gel electrophoresis with a gel concentration of 2% to detect the PCR products, and used the recovery kit to recover the products.

#### 2.3.4 Data analysis

The quality control of the original sequencing data was performed using FASTP (v0.20.0) (Chen et al., 2018). Splicing of the sequences was carried out using FLASH (v1.2.7) (Magoč and Salzberg, 2011). OTU clustering based on 97% similarity and removal of chimeras were conducted using UPARSE (v7.1) (Edgar, 2013). Each sequence was classified and annotated using RDP classifier (v2.2) with a comparison threshold set at 70%, against the Silva 16S rDNA database (v138) (Wang et al., 2007). Alpha diversity analysis was performed using Mothur (v1.30.2), while Beta diversity analysis based on Bray-Curtis distance was conducted in R language (v3.3). (v3.3.), employed principal component analysis (PCA), and the Kruskal Wallis H test was used for difference analysis. 16S and ITS sequencing were generated in Majorbio Co., Ltd. (Shanghai, China). Correlation analysis between rumen microbiota and SCFAs were performed using the Biozeron Cloud Platform (see text footnote 1).

### 2.4 Determination SCFAs of rumen fluid

The rumen fluid SCFAs concentration was determined by 7890B-5977B Gas Chromatography–Mass Spectrometry (GC–MS) (Agilent, GC System, U.S.). Specific analysis conditions are shown in Table 3.

**Table 3:** Determination parameters of chromatographic mass spectrometry.

Equipment	Specifications and conditions	Equipment	Specifications and conditions
Chromatographic column	CD-2560 (100 m×0.25 mm×0.25 μm)	Column temperature	60 °C for 2 min, 20 °C/min to 200 °C for 1 min
Sample volume	1 μL	Interface temperature	230 °C
Sample temperature	250 °C	Ion source temperature	230 °C
Split ratio	20:1	Quad temperature	150 °C
Carrier gas	Helium (99.999%)	Ionization mode	EI**+**, 70 ev
Flow	0.8 mL/min	Quality range	33~500

## 3 Results

### 3.1 The mRNA expression of TJP in tissues

**Figure 1:**
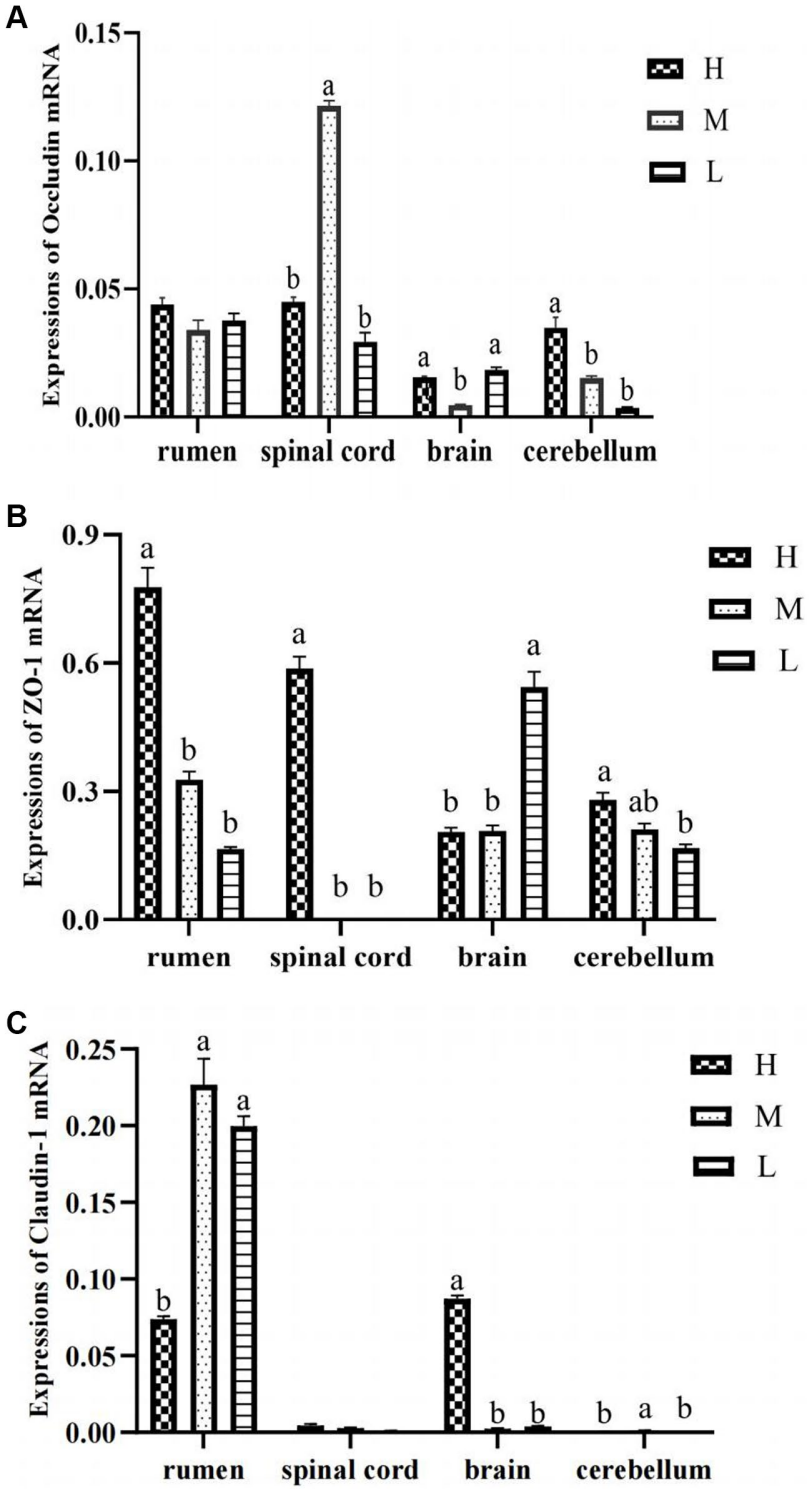
The mRNA expression of Occludin **(A)**, Claudin-1 **(B)**, and ZO-1 **(C)** in rumen epithelium, spinal cord, brain, and cerebellum.

As shown in Figure 1, when compared with group H, both groups M and L had increased mRNA expression of Claudin-1 in rumen epithelium but decreased ZO-1 expression (*p* < 0.05). The mRNA expressions of Occludin in spinal cord were reduced in group L compared with group H (*p* < 0.05). For brain tissue, group M down-regulated the mRNA expressions of Occludin and Claudin-1 when compared with group H (*p* < 0.05). Moreover, in the cerebellum the mRNA expressions of Occludin and ZO-1 in group H was lower than that in group L (*p* < 0.05).

### 3.2 Bacterial 16S rDNA and fungal ITS gene sequencing

In 16S rDNA sequencing, a total of 615,790 sequences were obtained from all samples (average 68,421 sequences per sample), and the average sequences in group H, M, and L were 70,358, 71,011, and 63,894, respectively (Table 4). The Chao index of group M was significantly higher than that of group L (*p* < 0.05). The Shannon index of group H was significantly lower than that of group L (*p* < 0.05). In ITS sequencing, the average sequences in group H, M, and L were 69,418, 67,219, and 73,521, respectively, the Shannon index of group H was significantly higher than that of groups M and L (*p* < 0.05).

**Table 4:** The analysis of α-diversity of bacteria and fungi in rumen of Honghe Yellow cattle.

Microorganism	Groups	Sequences	Shannon	Chao	Coverage
Bacteria	H	70358±1695	0.80±0.07^b^	16.00±1.15^ab^	0.99
	M	71011±1950	0.90±0.09^ab^	18.67±0.67^a^	0.99
	L	63894±3540	1.09±0.06^a^	15.33±0.67^b^	0.99
Fungi	H	69418±3484	1.11±0.07^a^	5.00±0.58	0.99
	M	67219±6106	0.70±0.11^b^	5.67±0.33	0.99
	L	73521±650	0.42±0.15^b^	5.33±0.88	0.99

No letters on the shoulder of peer data or the same letters indicate no significant difference (*p* > 0.05), and different lowercase letters indicate a significant difference (*p* < 0.05).

**Figure 2:**
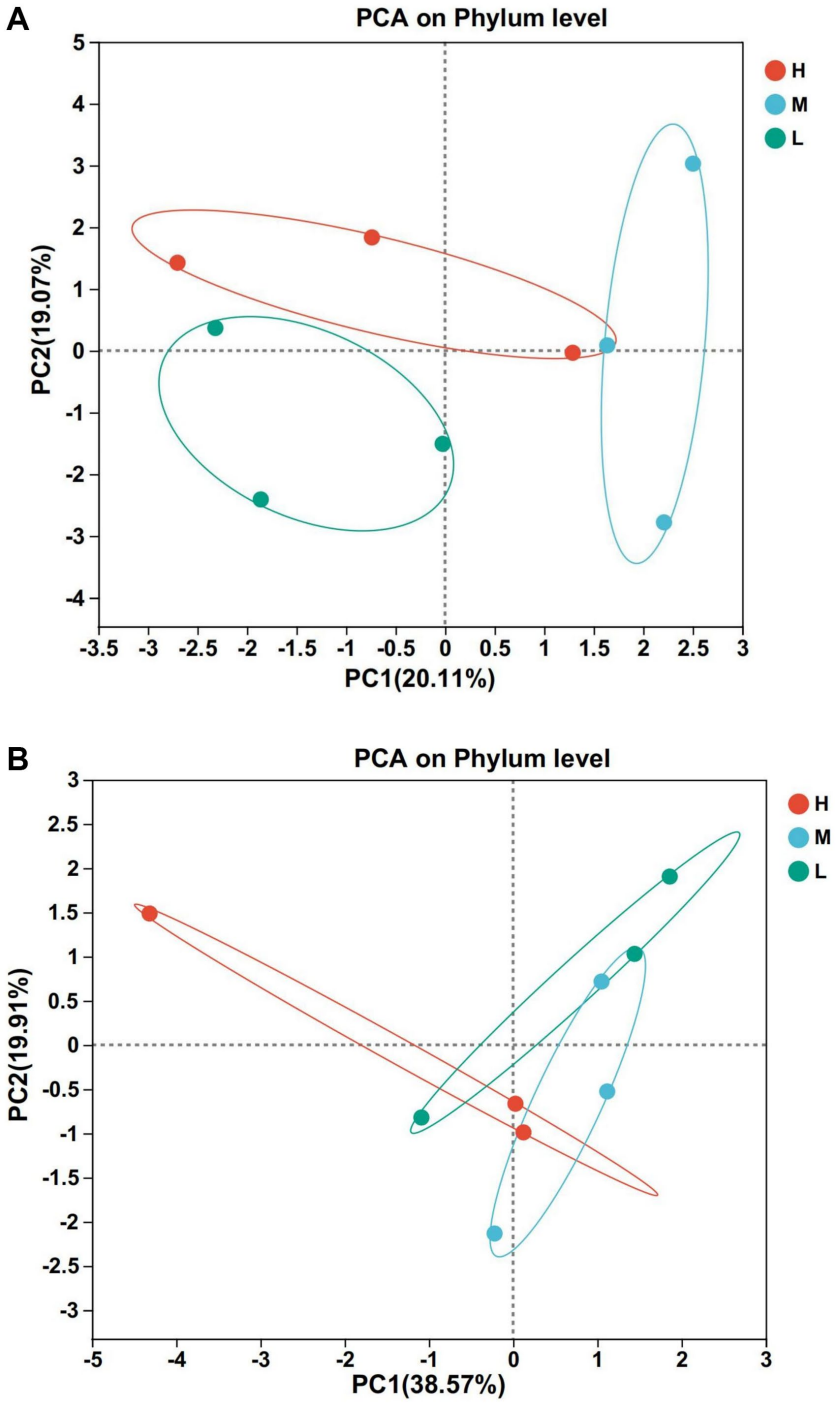
Principal component analysis (PCA). **(A)** Bacteria. **(B)** Fungi. Rows and columns represent the first principal component and the second principal component, respectively.

According to Figures 2A,B, there were differences in bacterial composition and structure at different energy diet levels, and the first two principal component scores accounted for 20.11 and 19.07% of the total variation, respectively. The composition and structure of fungi at different energy diets were different. The first two principal components scored 38.57 and 19.91% of the total variation, respectively.

### 3.3 Bacterial structure analysis and differential bacteria

**Figure 3:**
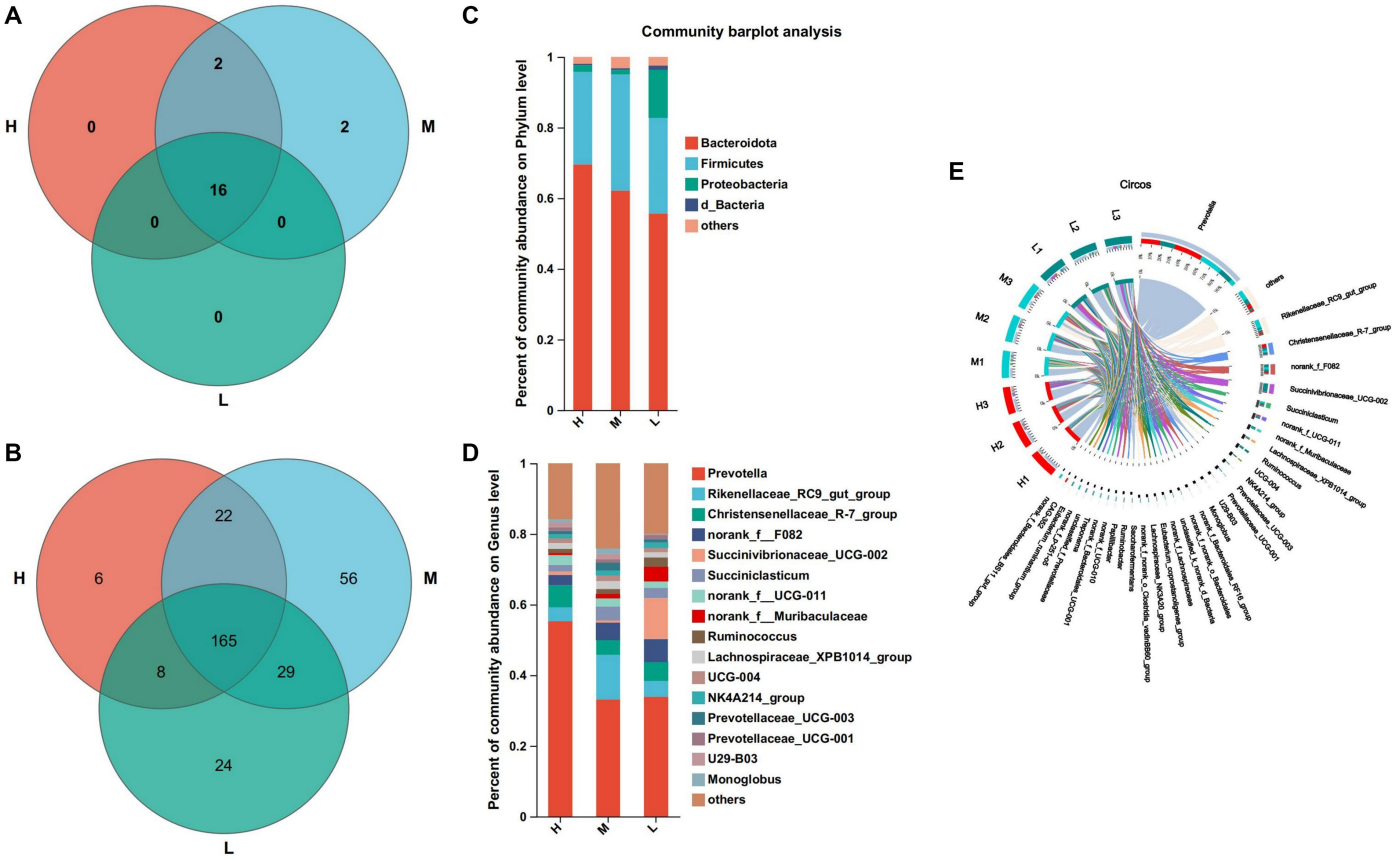
Composition and differential bacteria in the rumen. **(A)** Venn diagram at the phylum level. (C) Community structure at the phylum level. **(D)** Community structure at the genus level. **(B)** Venn diagram at the genus level. **(E)** Visualization Circle. Reflect the distribution proportion of dominant bacterial genera in the sample, as well as the distribution proportion of each dominant species in groups H, M and L.

At the level of 97% similarity, a total of 20 bacteria phyla were identified at the phylum level (Figure 3A). Among them, 16 bacteria phyla were shared by groups H, M, and L. Bdellovibrionota and Deinococotota were unique bacteria phyla of the group M, while Armatimonadota and Planctomycota were the common bacteria phyla of the groups H and M. Bacteroidota (H: 69.57%, M: 62.16% and L: 55.67%) and Firmicutes (H:26.18%, M: 32.88% and L: 27.12%) were the dominant phyla of the three groups (Figure 3C). At the genus level, 338 bacteria genera were identified (Figure 3B), including 165 bacteria genera shared by groups H, M, and L, 6 bacteria were unique to the group H, 56 bacteria were unique to the group M and 24 bacteria were unique to the group L (Figure 3B), *Prevotella* (55.33%) and *Christensenellaceac-R-7-group* (6.21%) were dominant bacteria in group H, Prevotella (33.17%) and *Rikenellaceae-RC9-gut-group* (12.58%) were dominant bacteria in group M, *Prevotella (33.89%)* and *Succinivibrionaceae-UCG-002* (11.6%) were dominant bacteria in group L (Figures 3D,E).

**Figure 4:**
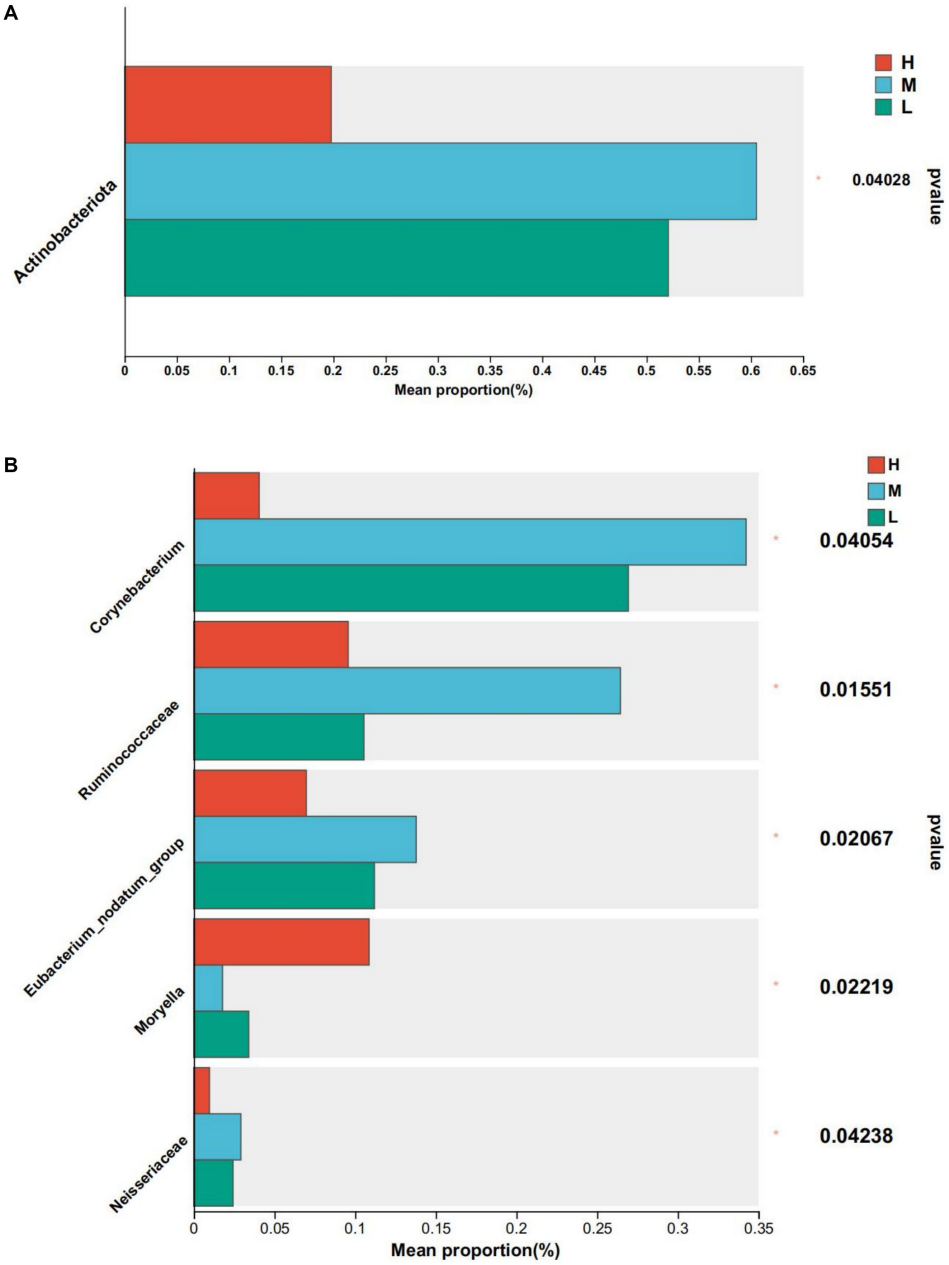
Differential bacteria, * means *p* < 0.05. **(A)** At phylum level. **(B)** At genus level.

At the phylum level, the abundance of actinomycetes was significantly higher in groups M and L compared to group H (Figure 4A). At the genus level, the relative abundance of *Corynebacterium*, *Eubacterium_nodatum_group*, and *Neisseriaceae* was significantly lower than that of Groups M and L (*p* < 0.05), the relative abundance of *Ruminococcaceae* was significantly lower than that of Group M (*p* < 0.05), and *Moryella* in Group H was significantly higher than that of Group M and Group L (*p* < 0.05) (Figure 4B).

### 3.4 Fungal structure analysis and differential fungi

**Figure 5:**
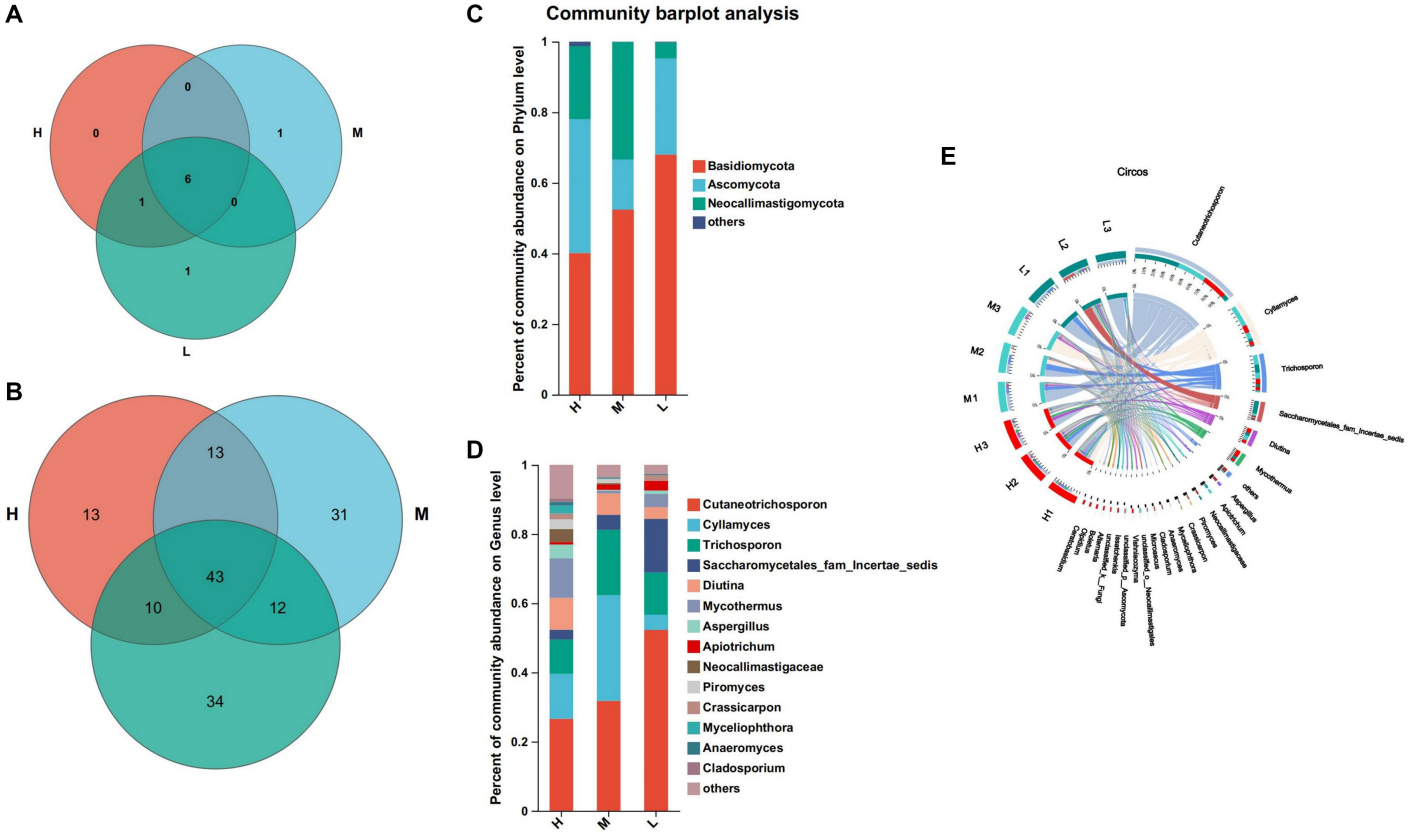
Composition and differential fungi in the rumen. **(A)** Venn diagram at the phylum level. **(B)** Venn diagram at the genus level. **(C)** Community structure at the phylum level. **(D)** Community structure at the genus level. **(E)** Visualization Circle. Reflect the distribution proportion of dominant fungal genera in the sample, as well as the distribution proportion of each dominant species in groups H, M and L.

At the phylum level, a total of 9 fungi phyla were identified by annotation (Figure 5A). Among them, 6 fungi phyla were common to three groups, Olpidiomycota was a common phylum of the groups H and L, Rozellomycota was a unique phylum of the group M, and Glomeromycota was a unique phylum of the group L. Basidiomycota (H: 40.11%, M: 52.40% and L: 67.99%), Ascomycota (H: 37.94%, M: 14.19% and L: 27.27%) and Neocariastigomycota (H: 20.64%, M: 33.31% and L: 4.51%) were the dominant fungi phyla in the three groups (Figure 5C). At the genus level, 156 fungi genera were identified, 43 fungi genera of which were common to three groups, 13 fungi genera were unique to the group H, 31 fungi genera were unique to the group M, and 34 fungi genera are unique to the group L (Figure 5B). The dominant fungi genera of the group H were *Cutaneotrichosporon* (26.59%), *Cylamyces* (13.00%) and *Mycothermus* (11.42%), the dominant fungi genera of the group M were *Cutaneotrichosporon* (31.77%), *Cylamyces* (30.56%) and *Trichosporon* (18.88%), the dominant fungi genera of the group L were *Cutaneotrichosporon* (52.3%) *Saccharomycetales-fam-Incertae-sedits* (15.50%) and *Trichosporon* (12.19%) (Figures 5D,E).

**Figure 6:**
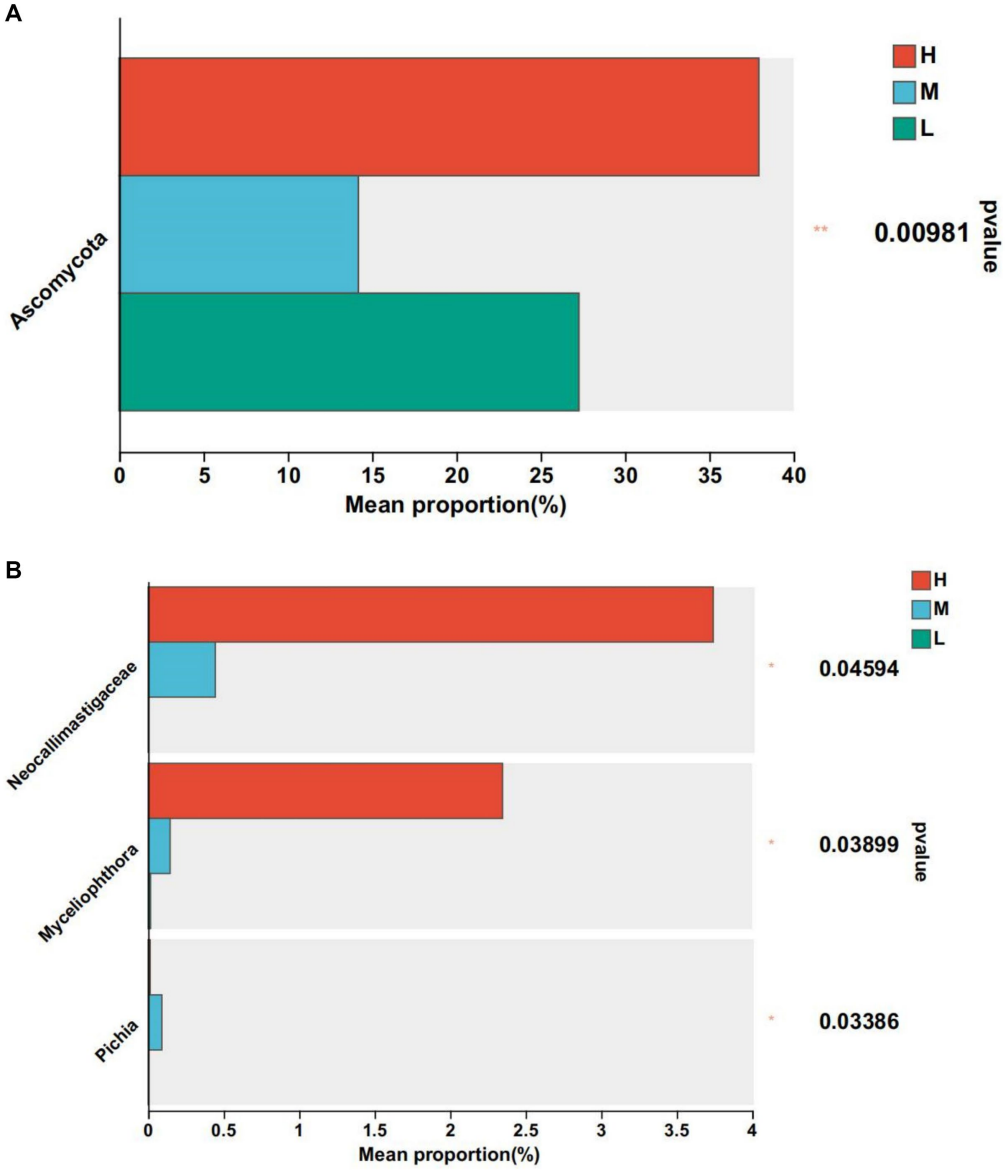
Differential fungi. * means *p* < 0.05. **(A)** At phylum level. **(B)** At genus level.

At the phylum level, Ascomycota was significantly higher than groups M and L compared to group H (*p* < 0.05). At the genus level, *Neocellimastigaceae*, *Myceliophthora* and *Pichia* were the differential fungal genera (Figure 6A). Compared with group H, the relative abundance of *Neocellimastigaceae* and *Myceliophthora* was higher than that of groups M and L (*p* < 0.05), and the relative abundance of *Pichia* was significantly lower than that of group M (*p* < 0.05) (Figure 6B).

### 3.5 SCFAs concentration in rumen fluid

According to Table 5, the concentration of SCFAs increased with decreasing dietary energy levels. In particular, when compared with group H, acetate content and A/P ritio (acetate to propionate ratio) were significantly lower (*p* < 0.05) than group L. The concentration of butyrate and isobutyrate concentrations were not significantly different (*p* > 0.05) among the three groups. The concentration of valerate and isovalerate were significantly higher in group M compared to group H (*p* < 0.05), and not significantly different from group L (*p* > 0.05).

**Table 5:** The concentration of SCFAs in rumen fluid of Honghe Yellow cattle.

Items	Group H	Group M	Group L
Total SCFA (mmol/L)	26.50±0.05^c^	27.43±0.09^b^	34.72±0.04^a^
Acetate (mmol/L)	18.26±1.30^b^	17.82±2.00^b^	26.87±0.74^a^
Propionate (mmol/L)	4.77±0.09^b^	7.24±0.33^a^	4.50±0.25^b^
Butyrate (mmol/L)	1.97±0.14	1.48±0.06	2.12±0.39
Isobutyrate (mmol/L)	0.72±0.08	0.53±0.03	0.61±0.06
Valerate (mmol/L)	0.23±0.03^a^	0.10±0.00^b^	0.20±0.05^ab^
Isovalerate (mmol/L)	0.55±0.07^a^	0.26±0.03^b^	0.42±0.05^ab^
A/P	3.84±0.31^b^	2.46±0.43^b^	5.97±0.28^a^

No letters on the shoulder of peer data or the same letters indicate no significant difference (*p* > 0.05), and different lowercase letters indicate a significant difference (*p* < 0.05).

### 3.6 Correlation of rumen microbiota with ruminal SCFAs and TJP

**Figure 7:**
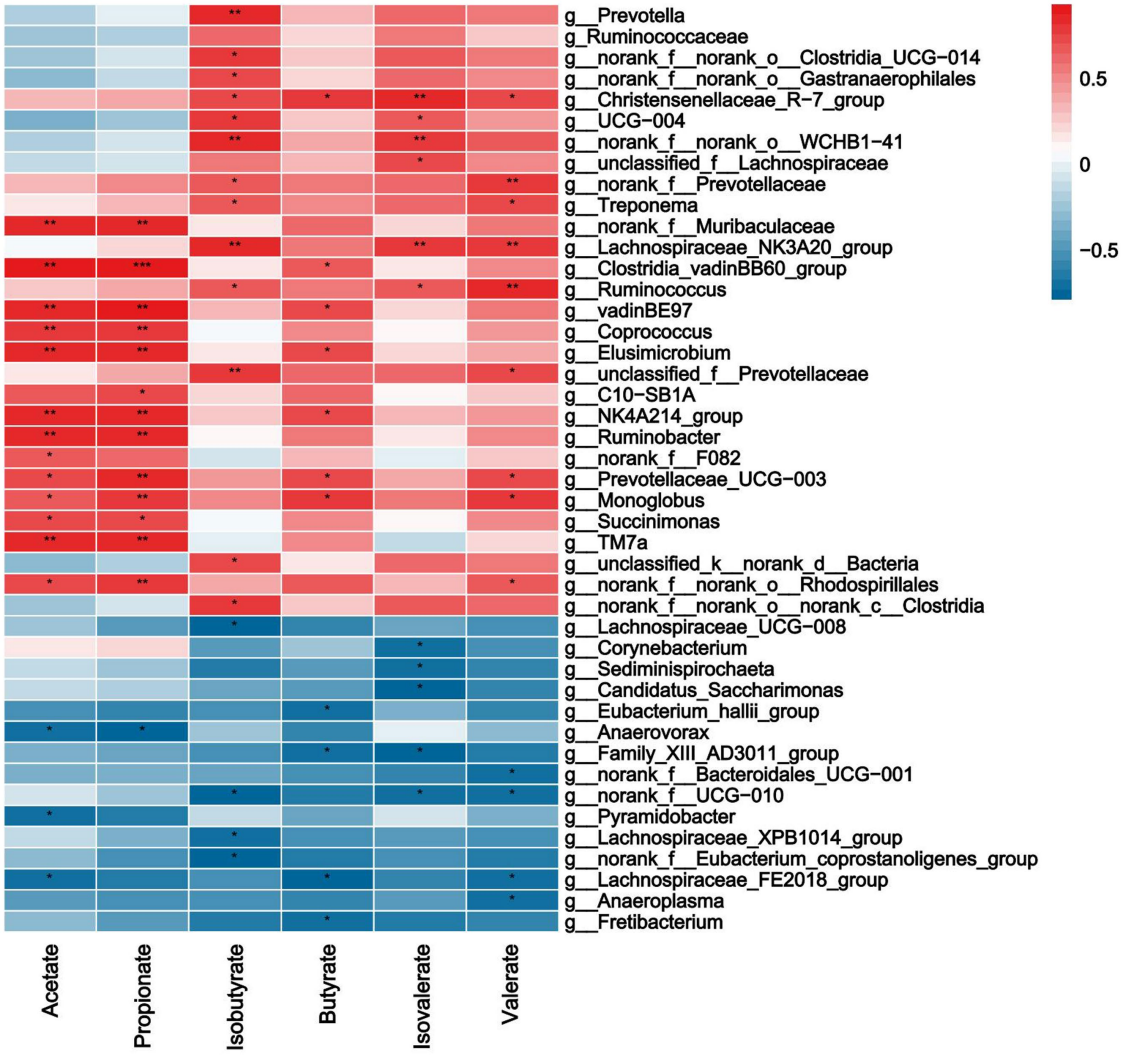
Correlations analysis between ruminal SCFAs and rumen bacteria. Rows and columns represent the indicated ruminal VFAs and genus, respectively. Each lattice represents a Pearson correlation coefficient between an indicator with a genus. Red and blue denote positive and negative correlation, respectively. * indicated the significant correlation, the more the correlation, the greater the similarity.

As shown in Figure 7, acetate, propionate was significantly positively correlated with *norank_F__Muribaculaceae*, *Clostridia_VadinBB60_Group*, *Coprococcus*, *Elusimicrobium*, *Rumnobacter*, *Prevotellaceae_UCG-003* and *Monolobus* (*p* < 0.0*5*), it was negatively correlated with *Anaerovora* etc. Isobutyrate was significantly positively correlated with *Prevotellaceae*, *Treponema*, *Lachnospiraceae_NK3A20_group*, *Ruminococcus*, etc., it was negatively correlated with norank_f__UCG − 010, *Lachnospiraceae_XPB1014_group* and *Eubacterium_coprostanoligenes_group* (*p* < 0.0*5*). Butyrate was significantly positively correlated with C*lostridia_vadinBB60_group, Elusimicrobium*, *Prevotellaceae_UCG − 003*, *Monoglobus,* etc., and it was negatively correlated with *Eubacterium_hallii_group* and *Family_XIII_AD3011_group*, etc. (*p <* 0.0*5*). Isovaleric was positively correlated with *Christensenellaceae_R − 7_group*, *Lachnospiraceae_NK3A20_group*, *Lachnospiraceae,* etc., there was a significant negative correlation between *Corynebacterium*, *Sediminispirochaeta* etc. (*p* < 0.0*5*).

**Figure 8:**
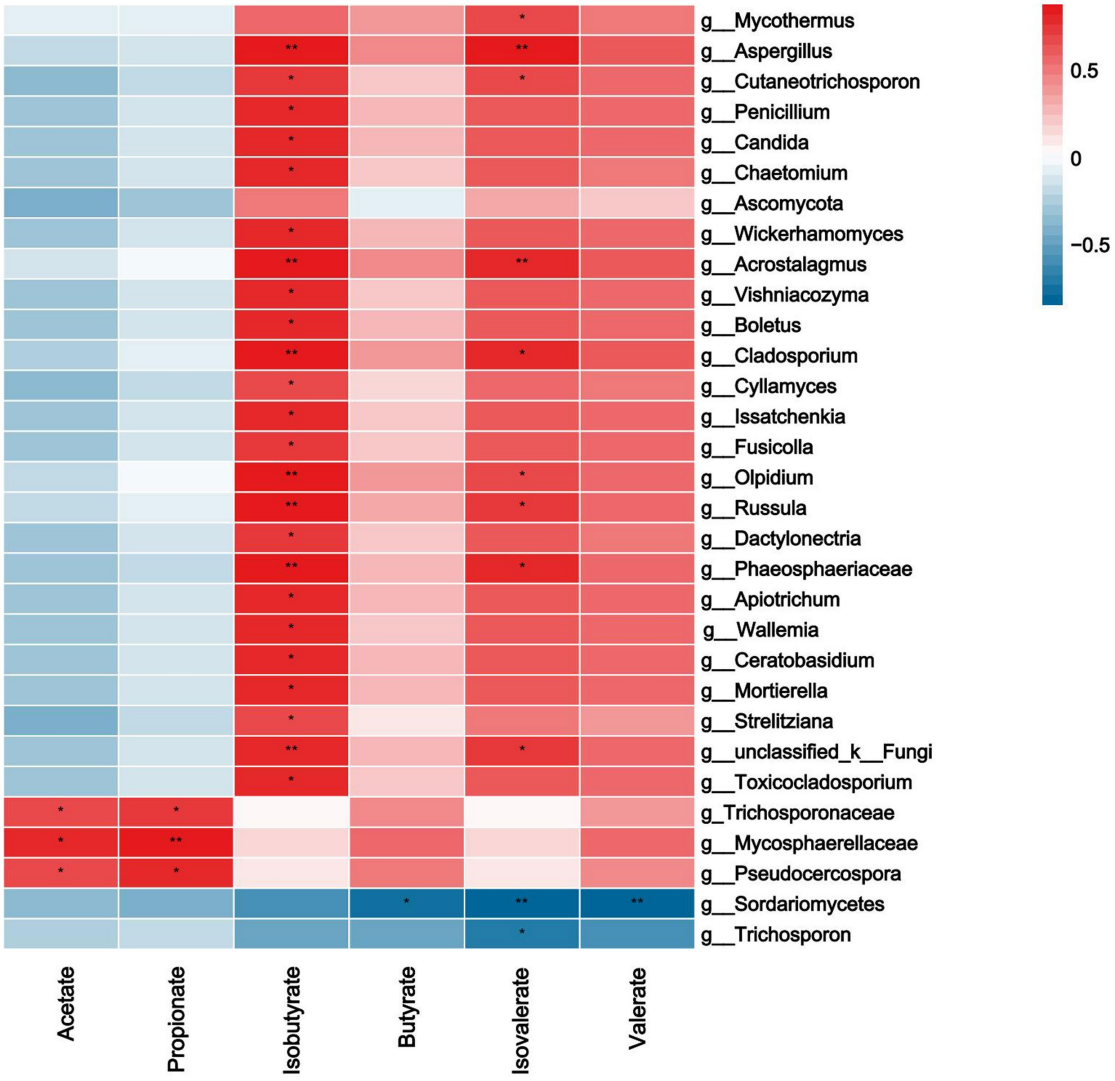
Correlations analysis between ruminal SCFAs and rumen fungi. Rows and columns represent the indicated ruminal SCFAs and genus, respectively. Each lattice represents a Pearson correlation coefficient between an indicator with a genus. Red and blue denote positive and negative correlation, respectively. * indicated the significant correlation, the more the correlation, the greater the similarity.

As shown in Figure 8, acetate and propionate were significantly positively correlated with *Trichosporonaceae, Mycosphaerellaceae, Pseudocercospora* (*p*<0.0*5*). Isobutyrate was significantly positively correlated with *Aspergillus, Cutaneotrichosporon and Penicillium* etc. (*p* < 0.0*5*). Butyrate was negatively correlated with *Sordariomycetes* (*p* < 0.0*5*). Isovaleric was positively correlated with *Mycothermus, Aspergillus and Acrostalagmus* etc., there was a significant negative correlation between *Sordariomycetes* and *Trichosporon* (*p* < 0.0*5*). Valerate was negatively correlated with *Sordariomycetes* (*p* < 0.0*5*).

**Figure 9:**
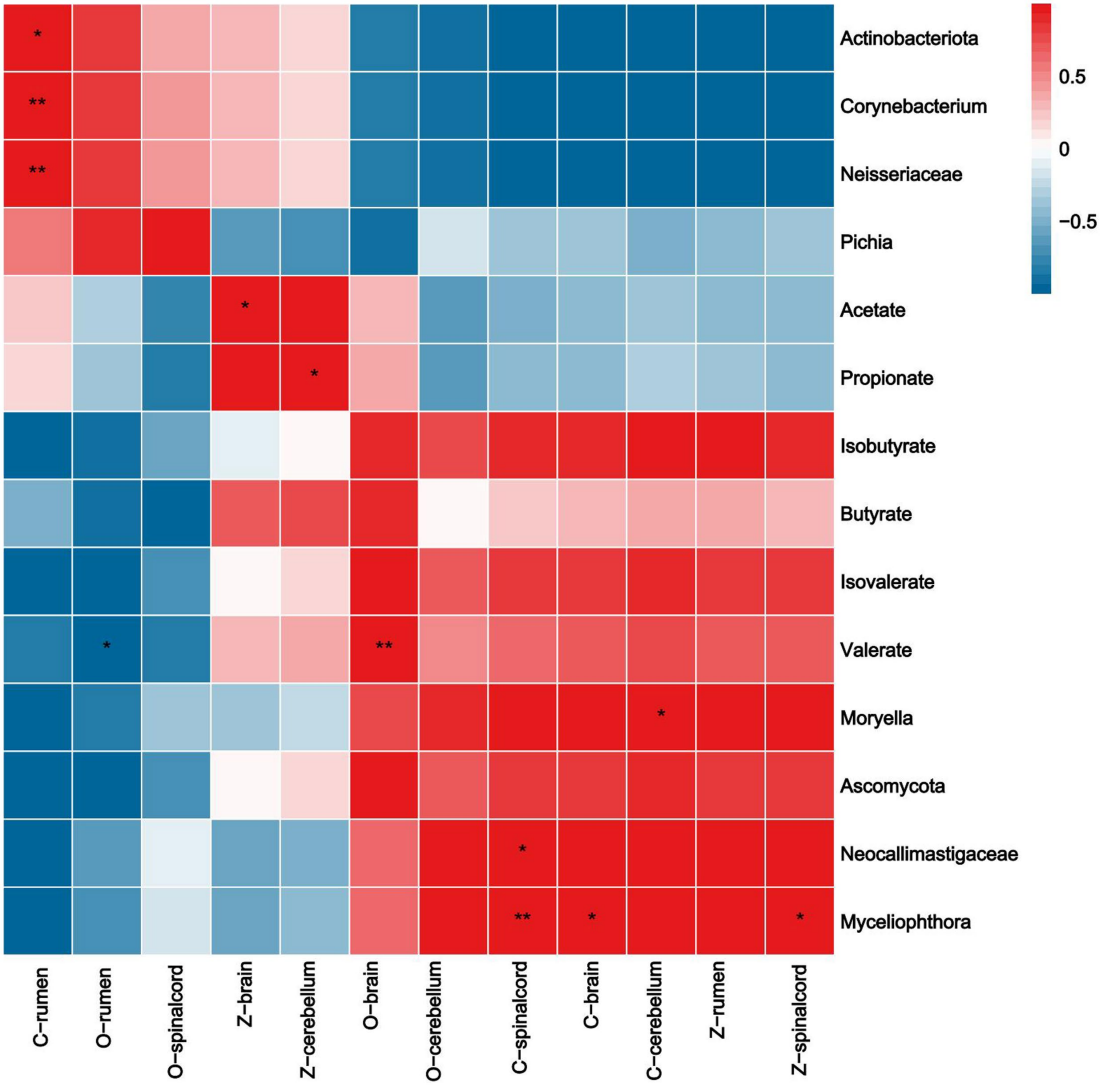
Correlation analysis of TJP with SCFAs and rumen differential bacteria, fungi. Rows represent the indicated expression of TJP in the rumen, brain, cerebellum, and spinal cord (O: Occludin, C: Claudin-1, Z: ZO-1), columns represent ruminal SCFAs and genus. Red and blue denote positive and negative correlation, respectively.

As shown in Figure 9, the expression of Claudin-1 in rumen epithelium was significantly positively correlated with *Actinobacteriota*, *Corynebacterium*, and *Neisseriaceae,* that in spinal cord was significantly positively correlated with *Neocallimastigaceae* and *Myceliophthora*, in brain with *Myceliophthora*, and in cerebellum with *Moryella.* The expression of ZO-1 in spinal cord was significantly positively correlated with *Myceliophthora*, and its relative expression in brain and cerebellum was positively correlated with the contents of acetate and propionate. Occludin expression in the brain was significantly positively correlated with valerate content, whereas expression in the rumen epithelium was significantly negatively correlated with valerate content.

## 4 Discussion

In this study, Claudin-1, Occludin, and ZO-1 were expressed in the brain, cerebellum, spinal cord, and rumen epithelium of the three groups, which is consistent with previous research results (Zihni et al., 2016). Claudin-1 had high relative expression in the rumen epithelium, Occludin had high relative expression in the rumen epithelium and spinal cord, and ZO-1 had high relative expression in the rumen epithelium and brain. TJP is an important form of intercellular junction and the most important structure constituting the mucosal mechanical barrier, which consists of a variety of structural proteins and various types of connecting protein molecules, which can mediate paracellular permeability and is responsible for intercellular transport and intestinal barrier permeability function (Dalile et al., 2019), which is confirmed by the high expression of the three TJP in the rumen epithelium. Among them, Occludin and ZO-1 had the highest expression in the rumen epithelium of fed animals with high energy diet, whereas Claudin-1 had the highest expression in the rumen epithelium of animals fed medium-energy diets, probably due to the fact that lower dietary energy levels are not conducive to the expression of tight junctional proteins. Feeding ruminants with a large number of high-energy diets can produce numerous nutrients such as SCFAs through fermentation of rumen microbiota (Rabelo et al., 2003). Moreover, rumen pH can be significantly reduced, osmotic pressure can also be significantly increased, and numerous harmful substances such as LPS, biogenic amines and acetaldehyde can be produced (Elmhadi et al., 2022). These harmful substances can damage cell connections and increase epithelial permeability (Saleem et al., 2012). This may be the reason for the low relative expression of Claudin-1 gene in the spinal cord and cerebellum, but the specific impact and mechanism need to be further studied.

Dietary energy plays an important role in rumen microbial diversity. In this study, Firmicutes and Bacteroides were the main dominant bacteria, which is similar to previous study (Kim et al., 2011). The energy feed in this study is mainly corn silage, which contains a lot of fiber. Fiber increases the available nutrients of Firmicutes and Bacteroides as the main component of the diet, which may be the reason why the abundance of these two bacteria is higher than that of other bacteria. Furthermore, in this study, the abundance of Bacteroides decreased with decreasing dietary energy levels. The abundance of Bacteroides is closely related to a diet with high protein and fat (Thompson et al., 2015). Firmicutes are closely related to cellulose decomposition (Petri et al., 2013). At present, we found that *Prevotella* was the dominant genus of the three groups, which is similar to the research results of Pitta et al. (2014). It was found that *Prevotella* contains members of protein hydrolysis, starch decomposition, and hemicellulose decomposition activities, and the main fermentation products are acetate, propionate and succinate (Rubino et al., 2017). In this study, the abundance of *Prevotella* in group H was the highest, which may be indicated that members of *Prevotella* related to protein, starch, and hemicellulose decomposition are involved in the digestion and utilization of high-energy diets.

The dominant fungi phylum of the three groups were Ascomycetes, Neoflagellates, and Basidiomycetes, which are similar to previous studies (Mao et al., 2016). The abundance of Basidiomycetes in group L was the highest, that of Neoflagellata in group M was the highest, and that of Neutrocystita in group H was the highest. This shows that a diet with a high energy level can promote the reproduction of ascomycetes, but inhibit the reproduction of Basidiomycetes. The total proportion of three fungi phyla in group M was the highest, reaching 99.9%, and that in groups H and L was 98.69 and 99.77%, respectively, indicating that the diet with medium energy level was more conducive to the growth and reproduction of fungi. The abundance of *Cutaneotrichosporon* increased gradually with the decreased of dietary energy level, indicated that low energy level diet is beneficial to the growth and reproduction of *Cutaneotrichosporon*. Ye et al. (2019) showed that the rumen fungi of Holstein cows in China were abundant in *Cladosporium*, *Aspergillus*, and *Dbaly*. The above results show that the dominant fungi with a higher relative abundance of different species are quite different at the genus level, this may be due to the differences in genes, diets, and growth environments of the three species.

The different microbial compositions will affect the SCFAs produced differently (Vacca et al., 2020). For example, Firmicutes mainly produce acetate, propionate and butyrate (Tagliabue and Elli, 2013), *Bacteroides* plays an important role in the production of acetate and propionate (Xue et al., 2017). *Clostridium* can produce propionate (Smith et al., 2013), *Prevotella* can produce propionate (Ramsak et al., 2000), and Trichospiridae can produce butyrate (Deusch et al., 2017). *Rikenellaceae-RC9*, *Christensenellaceae-R-7*, *norank-UCG-011*, and *Prevotellaceae-UCG-001* can also be used as substrates for acetate and propionate fermentation (Holman and Gzyl, 2019). At the present study, the abundance of Firmicutes and *Rikenellaceae-RC9* in group M was the highest, which led to the highest concentration of propionate. In group H, *Bacteroides* and *Christensenellaceae-R-7* had the highest abundance, and the concentrations of acetate and propionate were also high. In line with previous studies, the acetate level in the rumen was increased, the propionate level decreased by appropriately reducing the dietary energy level (Wang et al., 2020; Xie et al., 2021). In this experiment, the concentration of propionate in group M was the highest, which indicated that feeding the diet with a medium energy level to Honghe Yellow cattle can increase the content of propionate, reduce the proportion of A/P, and increase the content of gluconeogenesis.

Microbes and metabolites SCFAs can regulate the expression of TJP (Singh et al., 2019; Sochocka et al., 2019), and enhance the ability of bacteria to pass through the barrier (Bednarska et al., 2017), thus affecting the intestinal barrier function (Trindade et al., 2023). In this study, *Corynebacterium* was positively correlated with the relative expression of Occludin in the spinal cord and Claudin-1 in the rumen epithelium, while negatively correlated with the relative expression of Occludin and Claudin-1 in the brain, indicated their importance in the expression of TJP genes. However, the bacteria have opposite effects on the relative expression of the same TJP gene in different tissues, which may be due to the different dietary energy levels, the type of bacteria, and the specific expression of bacteria in different tissues. However, the lack of significant correlation between some bacteria and the TJP gene does not mean that these bacteria are not important, the specific mechanism by which bacteria interfere with TJP expression remains to be further investigated. SCFAs maintain intestinal barrier integrity and protect against intestinal inflammation (Lewis et al., 2010). SCFAs such as acetate, propionate and butyrate induced TJP expression, increased intercellular transport, and inhibited activation of inflammatory vesicles and autophagy. In a study of porcine intestinal barrier model, gavage of short-chain fatty acids enhanced the expression of tight junction proteins (Sivaprakasam et al., 2017). Among them, butyrate was specifically able to enhance intestinal barrier function by regulating the expression of tight junction proteins and was able to activate AMP-activated protein kinase (AMPK), which in turn promoted intestinal barrier integrity by facilitating TJP assembly (Wang et al., 2012). Butyrate inhibits the expression of Claudin-2, which strengthens the intercellular TJP structure, inhibits intestinal permeability, and enhances the barrier defence function (Zhao et al., 2020), which may be the reason for the high expression of ZO-1 and Occludin in group H. Butyrate promotes the intestinal barrier function at low concentrations, and may impair the intestinal barrier function at high concentrations (Feng et al., 2018). Valerate and isovalerate showed a negative correlation with the relative expression of Claudin-1 in the rumen epithelium and a positive correlation with the relative expression of Claudin-1 in the brain, which may be caused by the dietary energy level and the specific expression of Claudin-1 in different tissues. Although an increasing number of studies in recent years have led to a preliminary knowledge and understanding of the role of SCFA in the expression of TJP outside of intestinal tissues, questions remain about the complex communication between the two and more experiments are needed to further reveal the role played by SCFAs.

## 5 Conclusion

The results indicated dietary energy levels influenced the community structure of bacteria, fungi and SCFAs concentration in rumen, as well as the mRNA expression of TJP in the tissues of Honghe Yellow cattle. The mRNA expression of Claudin-1 in rumen epithelium and the total SCFAs concentration were increased with decreasing dietary energy levels, then the mRNA expression of Claudin-1 in brain and ZO-1 in the spinal cord were reduced with decreasing dietary energy levels. In addition, TJP was significantly correlated with rumen microbiota and the content of SCFAs. Future studies need to further explore the mechanisms of interaction between rumen microbiota, SCFAs and barrier function.

## Data availability statement

The datasets presented in this study can be found in online repositories. The names of the repository/repositories and accession number(s) can be found at: NCBI – PRJNA1080663.

## Ethics statement

The animal study was approved by Yunnan Agricultural University. The study was conducted in accordance with the local legislation and institutional requirements.

## Author contributions

YY: Conceptualization, Data curation, Formal analysis, Investigation, Methodology, Writing – original draft. YZ: Formal analysis, Methodology, Writing – review & editing. RF: Data curation, Formal analysis, Investigation, Writing – original draft. BF: Formal analysis, Methodology, Writing – review & editing. CL: Methodology, Writing – review & editing. YL: Formal analysis, Methodology, Writing – original draft. ZL: Data curation, Writing – original draft. HW: Writing – review & editing. JL: Conceptualization, Funding acquisition, Investigation, Methodology, Project administration, Supervision, Validation, Writing – review & editing.

## Footnotes

^1^http://www.cloud.biomicroclass.com/CloudPlatform
